# Management of an Infected Vesicourachal Diverticulum in a 42-Year-Old Woman

**DOI:** 10.1155/2020/8886936

**Published:** 2020-08-02

**Authors:** Maria Erodotou, Maria Isaia, Georgios Fragkiadakis, Theocharis Tontikidis, Kosmas Tyriakidis, Spyridon Palaiologos, Nikolaos Nikolaou

**Affiliations:** ^1^General Surgery Department, Larnaca General Hospital, Larnaca, Cyprus; ^2^Urology Department, Larnaca General Hospital, Larnaca, Cyprus

## Abstract

Urachal remnant anomalies are uncommon in adults and can be confused with a variety of clinical conditions when symptomatic or infected. Vesicourachal diverticulum is the rarest type, accounting for approximately 3% to 5% of congenital urachal anomalies. We report the case of a 42-year-old female patient, who presented to the emergency department with lower abdominal pain and a palpable abdominal mass. An infected vesicourachal diverticulum was diagnosed after imaging studies and was initially treated with intravenous antibiotic therapy and drainage of the urachal diverticulum to the urinary bladder through a JJ stent. Finally, the patient underwent open surgical excision of the urachal remnant. The postoperative course was uneventful, and the histopathological examination confirmed the diagnosis of vesicourachal diverticulum. We recommend drainage of an infected vesicourachal diverticulum through the bladder by JJ stent placement inside its lumen during cystoscopy, as an alternative to percutaneous drainage reported in the literature.

## 1. Introduction

Urachal remnant anomalies are rare in adulthood, with a reported incidence of 1 : 5000 in adults, and a higher prevalence in men than women with a ratio 2 : 1 [1, 2]. The urachus is an embryologic remnant of the cloaca and the allantois [1], and incomplete obliteration of its lumen results in four different types of urachal anomalies [1, 2]. Vesicourachal diverticulum, where there is a persistence of the vesical portion of the urachus, is the most uncommon, accounting for approximately 3% to 5% of congenital urachal anomalies [3]. It is generally asymptomatic and discovered incidentally at imaging studies [1, 3]. Infection is the most common complication of urachal remnants and may present with nonspecific abdominal or pelvic symptoms. When urachal diverticula become symptomatic or infected, surgical management is indicated [1, 3].

## 2. Case Presentation

**Figure 1:**
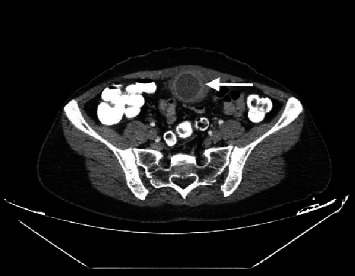
Contrast–enhanced computed tomographic image showing a cystic lesion with peripheral enhancement (arrow), thus excluding the initial diagnosis of an incarcerated hernia.

**Figure 2:**
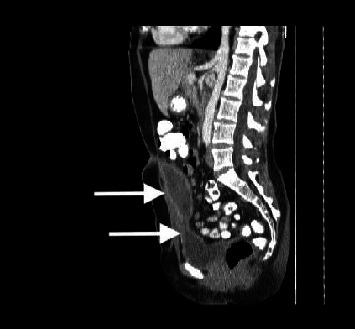
Contrast–enhanced computed tomography (sagittal plane) showing the cystic lesion (arrow-above) arising from the bladder dome (arrow–below) and extending to the umbilical region, leading to a suspected diagnosis of an infected vesicourachal diverticulum.

**Figure 3:**
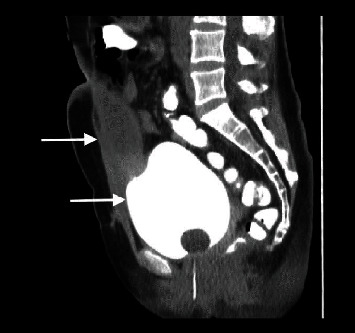
Cystography showing the bladder filled with contrast (arrow-below) without diffusion of the contrast inside the vesicourachal diverticulum (arrow-above), probably due to infection and a small opening of its lumen.

**Figure 4:**
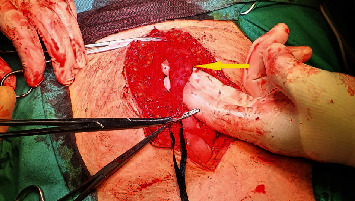
Intraoperative image during the dissection of the urachal remnant (arrow).

**Figure 5:**
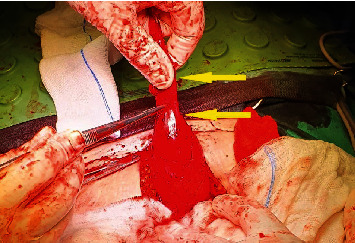
Intraoperative image showing the vesicourachal diverticulum (arrow-above) arising from the anterosuperior margin of the bladder (arrow-below).

**Figure 6:**
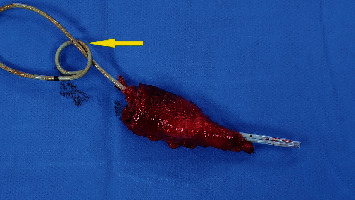
Surgical specimen of the resected vesicourachal diverticulum with a bladder cuff and the JJ stent in place (arrow).

A 42-year-old woman presented to the emergency department complaining of progressively deteriorating lower abdominal pain of 2 days duration. Her past medical history was unremarkable, and hysterectomy and appendectomy were reported from her surgical history. On physical examination, diffuse tenderness in the lower abdomen and a palpable midline suprapubic mass were found. An incarcerated incisional hernia was suspected initially. Her vital signs were stable. Laboratory tests revealed leucocytosis of 13.5 × 10^9^/L with 77.2% neutrophil predominance and a high C-reactive protein of 93.4 mg/L. Urine analysis was normal and urine culture showed no bacterial growth. Abdominal ultrasonography (US) revealed a cystic lesion extending from the umbilicus to the bladder dome, measuring 7.8 × 3.5 cm, and free fluid in the pouch of Douglas. Computed Tomography (CT) revealed a cystic lesion with peripheral enhancement arising from the bladder dome and extending to the umbilical region, leading to a suspected diagnosis of an infected urachal diverticulum (Figures 1 and 2). A cystography was additionally performed which also revealed findings compatible with an infected urachal diverticulum (Figure 3). The initial treatment included empirical intravenous broad-spectrum antibiotic therapy with ciprofloxacin 400 mg twice daily. Cystoscopy revealed the orifice of the infected vesicourachal diverticulum, which was narrow with purulent content, so a JJ stent was placed through for drainage. Three months later, the patient underwent a scheduled open surgical excision of the urachal remnant including a cuff of normal bladder, through an infraumbilical vertical midline incision (Figures 4–6). The postoperative course was uneventful, and the patient was discharged on the 9th postoperative day with the urinary catheter in place. On a follow-up visit at two weeks, the catheter was removed, and the patient remained asymptomatic. The histopathological examination confirmed the diagnosis of vesicourachal diverticulum.

## 3. Discussion

The urachus, or median umbilical ligament, is a midline ductal remnant of the allantois and the cloaca that connects the anterosuperior bladder to the umbilicus. This fibrous remnant is located extraperitoneally in the space of Retzius (or retropubic space), between the transverse fascia anteriorly and the parietal peritoneum posteriorly. It varies from 3 to 10 cm in length, and it commonly has an approximate diameter of 8 to 10 mm [1–3]. Histologically, the urachus is composed of three layers, an inner layer lined with transitional epithelium in 70% of cases and with columnar epithelium in 30%, a middle submucosal layer of connective tissue, and an outermost muscular layer in continuum with the detrusor muscle. However, urachal remnants without an epithelial lining have also been described [1, 3, 4].

The urachus involutes before birth and remains as a connective tissue band, the median umbilical ligament. Incomplete obliteration of the urachus results in four types of congenital anomalies such as patent urachus (50%) in which a communication between the bladder and the umbilicus exist, umbilical-urachal sinus (15%) in which the urachus opens into the umbilicus and drainage from the umbilicus can be present, vesicourachal diverticulum (3-5%) in which the urachus has a wide patent opening into the bladder, and urachal cyst (30%) in which the urachus encompasses a cyst-like lesion within its length [3, 5]. Patent urachus is purely congenital, while the other types may close normally after birth but reopen in association with conditions that are often categorized as acquired diseases. These include infection and neoplasm [3]. With the widespread use of imaging, more asymptomatic cases are identified, and according to a more recent study, urachal cysts are the most common type, accounting for 69% [1].

Urachal remnants may remain asymptomatic, but when complicated by infection they may be confused with other abdominal or pelvic diseases at clinical examination or with malignant tumors at imaging, due to nonspecific symptoms [1, 3]. Vesicourachal diverticula are generally asymptomatic, as they usually have a large opening and drain well into the bladder, decreasing the possibility of complications [1]. However, a small opening can lead to debris collection or intraurachal stone formation from intermittent occlusion of the lumen, resulting in the clinical presentation of urinary tract infections, palpable mass, or acute abdominal pain if ruptured [6]. An increased prevalence of carcinoma after puberty is also reported in association with vesicourachal diverticula [3]. In the present case, cystoscopy revealed a small opening of the diverticulum to the bladder, which was blocked leading to infection. This was also demonstrated by the CT cystography where no contrast material was diffused inside the diverticulum lumen.

The urachus is located in a clinically silent area, so the clinical signs in case of infection or neoplasm may be nonspecific, delayed, or absent [5]. Patients with infection may present with abdominal pain with tenderness and palpable mass, as in the present case, fever, erythema, purulent urinary discharge, and dysuria or as an acute abdomen [1, 5]. Laboratory tests may show signs of inflammation such as elevated sedimentation rate or leukocytosis. On urine analysis, bacteriuria and pyuria are absent in more than 80% and urine culture is negative [5], as in the present case. Consequently, infected urachal remnants can be confused with a wide spectrum of diseases, such as complicated Meckel's diverticulum, acute prostatitis, acute appendicitis, ovarian torsion, endometriosis, recurrent urinary tract infections, abdominal colicky pain of unknown origin, or incarcerated hernia [1, 5]. In a reported case, presentation and physical examination suggested an incarcerated umbilical hernia as the predominant pathology, before imaging studies revealed an infected urachal cyst [7]. Similarly, in the present case, an incarcerated incisional hernia was suspected initially.

Abdominal US, CT scan, MRI, cystography, sinography, and cystoscopy can help with the differential diagnosis and they can show the relationship of the infected urachal anomaly with the surrounding tissue and adjacent organs [1]. Imaging findings such as complex echogenicity at US and heterogenous attenuation with variable contrast enhancement at CT scan make it difficult to differentiate an infected urachal remnant from urachal carcinoma. *Ι*n most of the cases, percutaneous needle biopsy or fluid aspiration may be necessary for the diagnosis and therapeutic planning [3]. However, the presence of hematuria, palpable suprapubic mass, and calcifications on CT (70% of cases) is diagnostic for urachal carcinoma [1, 4]. Urachal carcinomas are rare accounting for less than 1% of bladder cancers [1] and are typically silent because of their extraperitoneal location, with a poor prognosis due to late presentation and advanced disease with local invasion [1, 3].

The initial management of infected urachal anomalies should include broad–spectrum antibiotic therapy, combined with percutaneous or surgical drainage. Total surgical excision should be performed after the infection has cleared [1]. This two-stage approach is considered by most as the treatment of choice [2, 5]. It emphasizes infection resolution before surgical intervention as superior, for reducing the risk of postoperative complications, like wound infection or urine leak, as well as reducing hospital stay [7]. However, the primary excision of the urachal remnant before the inflammation subsides has also been reported, and it can be considered safe in cases of isolated urachal cyst infection [5, 7]. The two-stage approach may be more appropriate in the management of cases complicated by bladder fistula, large abscesses, and sepsis [7]. The present case was considered as complicated because of the communication with the bladder, therefore antibiotic therapy and drainage were implemented as a first step. However, we did not perform percutaneous or surgical drainage as described in the literature [1, 5]. The infected vesicourachal diverticulum was drained through the bladder, by placing a JJ stent inside its lumen during cystoscopy. To our knowledge, nothing similar is described in the literature. We consider the placement of a JJ stent as a simple, safe, and effective technique for the drainage of an infected vesicourachal diverticulum with a narrow opening to the bladder.

The recommended surgical approach for all the types of urachal anomalies is complete excision of the urachal remnant, in order to avoid the 30% risk of infection recurrence and the potential of malignant transformation later in life [1]. Anomalies that extend to the bladder, like patent urachus and vesicourachal diverticulum, additionally require resection of a cuff of normal bladder. This radical excision requires removing the urachus and each medial umbilical ligament, along with the adjacent peritoneum from the umbilicus to the bladder dome [1, 5]. In the traditional open surgical technique, an infraumbilical transverse or vertical midline incision is used [1, 5]. Laparoscopic surgery is a safe and effective technique, alternative to open surgery in the management of urachal remnants, with the advantages of minimal invasiveness [2, 5]. Robotic-assisted approach has also been reported [8].

## 4. Conclusion

Vesicourachal diverticulum is the most uncommon type of urachal remnant anomalies. Although rarely detected and even more rarely presenting with symptoms of infection, it should be considered in the differential diagnosis of cases with abdominal pain. Because of nonspecific symptoms at presentation, imaging studies like ultrasound, computed tomography, and cystoscopy are essential for final diagnosis. The initial management of infected urachal diverticula should include antibiotic therapy combined with adequate drainage. We would like to add drainage through the bladder by JJ stent placement inside the diverticulum as an alternative to percutaneous drainage reported in the literature. The total excision of the urachal remnant, including resection of a cuff of normal bladder, consists of the final treatment, in order to prevent infection recurrence or future malignancy.
